# The Combined Extract of *Zingiber officinale* and *Zea mays* (Purple Color) Improves Neuropathy, Oxidative Stress, and Axon Density in Streptozotocin Induced Diabetic Rats

**DOI:** 10.1155/2015/301029

**Published:** 2015-04-12

**Authors:** Jintanaporn Wattanathorn, Paphaphat Thiraphatthanavong, Supaporn Muchimapura, Wipawee Thukhammee, Kamol Lertrat, Bhalang Suriharn

**Affiliations:** ^1^Department of Physiology, Faculty of Medicine, Khon Kaen University, Khon Kaen 40002, Thailand; ^2^Integrative Complementary Alternative Medicine Research and Development Center, Khon Kaen University, Khon Kaen 40002, Thailand; ^3^Faculty of Agriculture, Khon Kaen University, Khon Kaen 40002, Thailand

## Abstract

Based on the protective effect of the combined extract of purple waxy corn and ginger (PWCG) on oxidative stress related disorders in diabetic condition, we aimed to determine the effect of PWCG on the functional, biochemical, and structural change of the lesion nerve in streptozotocin- (STZ-) diabetic rats. PWCG at doses of 100, 200, and 300 mg·kg^−1^ BW were orally given to STZ-diabetic rats which were subjected to chronic constriction (CCI) at right sciatic nerve for 21 days. The blood sugar was assessed before and at the end of study whereas the sciatic function index (SFI), paw withdrawal threshold intensity (PWTI), and paw withdrawal latency (PWL) were assessed every 3 days until the end of study. At the end of study, the determination of nerve conduction velocity (NCV), axon density, oxidative stress status, and aldose reductase (AR) activity of the lesion nerve were performed. It was found that PWCG improved SFI, PWTI, PWL, and NCV together with the improved oxidative stress status and the axon density in the lesion nerve. No changes of AR activity or blood sugar level were observed. Therefore, PWCG might improve the functional and structural changes in STZ-diabetic rats plus CCI via the improved oxidative stress status.

## 1. Introduction

Diabetic neuropathy (DN), a common and costly complication of diabetic mellitus [1], affects more than 50% of diabetic patients [2]. It has been reported that chronic hyperglycemia can destroy sensory, motor, and autonomic fibers especially at distal extremities [3]. The manifestation of DN is varied depending on the affected nerve ranging from sensory deficit, weakness, and autonomic dysfunction. DN is regarded as a leading cause of nontraumatic amputation [4] due to the loss of protective limb mechanical sensations. In addition, approximate 11% of DN cases are associated with chronic painful condition which markedly decreases the quality of life of diabetic patients [5]. Since diabetic condition is dramatically increased worldwide, the importance of DN is also increasing. However, the current therapeutic efficiency is still limited.

Recently, it has been demonstrated that hyperglycemia can induce nerve damage by many mechanisms such as the enhanced oxidative stress, the increase of advanced glycation end product formation [6–8], the accumulation of sorbitol due to the increased aldose reductase activity [9], and nerve hypoxia/ischemia [6–8]. However, accumulative lines of evidence have suggested that both the excess oxidative stress and the elevation of aldose reductase may possibly be the important cause for the development of DN. This raises the hypothesis that substances which can suppress oxidative stress and aldose reductase should improve DN.

Herbal medicine is used for treating numerous ailments on the long term. It has been believed that it is much safer than synthetic drugs. Therefore, herbal medicine is very popular and gains much attention nowadays. To decrease the high medical cost and expenditure, the dietary therapy by using herb-based food has gained much attention. Recently, it has been reported that* Zingiber officinale* and* Zea mays* (purple color) could improve diabetes and diabetic conditions [10–12]. In addition, recent study of our group has clearly shown that the combined extract of* Z. officinale* and* Z. mays* (purple waxy corn var. purple color) significantly improves diabetic cataract and diabetic retinopathy by suppressing oxidative stress and aldose reductase [13]. Therefore, the beneficial effect of the combined extract of purple waxy corn and ginger (PWCG) might be able to improve DN in streptozotocin- (STZ-) diabetic rats. Due to the lack of supported evidence, this study was undertaken to determine the effect of PWCG on DN in STZ-diabetic rats. In addition, the effects of PWCG on oxidative stress markers, aldose reductase activity, and axonal change were also investigated.

## 2. Materials and Methods

### 2.1. Plant Material and Extract Preparation

**Figure 1:**
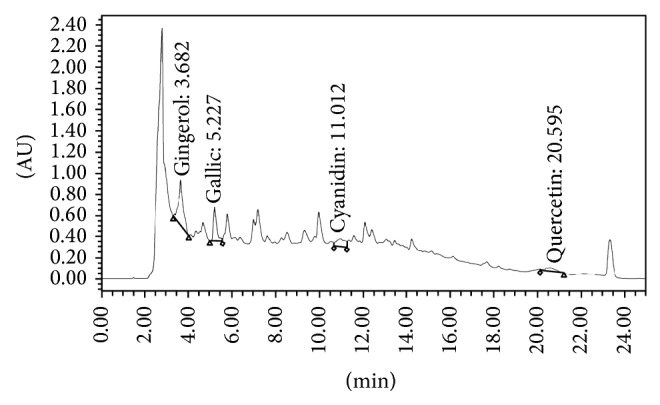
Fingerprint chromatogram of the combination extract of purple waxy corn (*Z. mays *var. purple color) and ginger (*Z. officinale* rhizome) or PWCG.

The plant materials used in this study were dried seeds of purple waxy corn or* Zea mays* L. (purple color; KKU open pollinated cultivar) and rhizomes of ginger or* Zingiber officinale* Roscoe. They were harvested during September 2012 and authenticated by Associate Kamol Lertrat and Dr. Bhalang Suriharn, Faculty of Agriculture, Khon Kaen University, Khon Kaen, Thailand. The voucher specimens (voucher specimens 2012001 and 2012002) were kept at the Integrative Complementary Alternative Medicine Research and Development Center, Khon Kaen University. The dried seeds of purple waxy corn and rhizomes of ginger were extracted with 50% hydroalcoholic solvent by maceration method at a ratio of 2 : 5 and 1 : 5 (weight : volume), respectively. The samples were macerated at room temperature for 3 days. Both of the yielded extracts were concentrated by lyophilization and kept at 4°C for further study. The percentage yields of purple waxy corn and ginger extracts were 5.72 and 25.26, respectively. The combination extract was prepared by mixing the extracts of purple waxy corn and ginger at a ratio of 1 : 4 (this ratio provided the highest anticataract potential). The content of total phenolic compounds in the combination extract was 44.82 ± 2.37 mg/L GAE/mg extract. The extract contained quercetin, gingerol, anthocyanin, and gallic acid at concentrations of 14.421 mg quercetin equivalent (QE)/100 mg PWCG extract, 10.701 mg gingerol/100 mg PWCG extract, 0.089 mg cyanidin-3-glucoside/100 mg PWCG, and 0.022 mg gallic acid/100 mg PWCG, respectively. The fingerprint chromatogram was shown in Figure 1.

### 2.2. Experimental Design

Male Wistar rats, weighing 250–280 g, were used in this study (*n* = 8 per group). The animals were maintained and treated in accordance with the guideline and approval of the Ethical Committee on Animals Experiments of Khon Kaen University (AEKKU 98/2555). All rats were divided into various groups as the following:

 group I: control group (all rats in this group were normal rats which are subjected to sham operation and received citrate buffer, a vehicle of streptozotocin (STZ)),
 group II: DM + CCI + vehicle of the extract or distilled water,
 group III: DM + CCI + GABApentin at dose of 50 mg·kg^−1^ BW,
 group IV: DM + CCI + ascorbic acid at dose of 100 mg·kg^−1^ BW,
 groups V–VII: DM + CCI + PWCG at doses of 100, 200, and 300 mg·kg^−1^ BW, respectively.

In all rats in groups II–VII, diabetes mellitus was induced by a single injection of STZ which was dissolved in citrate buffer (pH 4.5) at dose of 55 mg·kg^−1^ BW. All diabetic rats which showed the blood sugar level more than 250 mg·dL^−1^ were selected for further study by inducing peripheral neuropathy with neuropathic pain by using the chronic constriction injury (CCI) of sciatic nerve [14, 15]. All rats were treated with the assigned interventions once daily after chronic constriction injury throughout the 21-day study period. The behavioral tests including hot plate test, von Frey filament, and foot print analysis were evaluated every 3 days until the end of study. At the end of study, nerve conduction velocity was investigated and the lesion nerves were evaluated upon the following parameters: histomorphology, aldose reductase, and oxidative stress markers including malondialdehyde (MDA) level and the activities of superoxide dismutase (SOD), catalase (CAT), and glutathione peroxidase (GPx) enzymes.

### 2.3. Induction of Diabetic Neuropathy: Chronic Constriction Injury

Diabetic neuropathy was induced as previously described [14, 15]. After the anesthesia with ethyl ether, the right hind limb was immobilized in extended and slightly elevated position. The biceps femoris muscle was exposed and the femur bone was used as a landmark for direction of incision. The silk thread number 4.0 was slipped under the sciatic nerve trifurcation and ligated. After the nerve ligation, the muscle and skin layers were sutured. Each animal was allowed to recover for 24 hours before the investigation was carried out further. In this experiment, the contralateral thighs of all treated groups were not subjected to the operation.

### 2.4. Determination of Walking Track Analysis

Since walking track analysis is one of the commonly used tools to assess the function of innervated target organs after nerve injury, it was used as the evaluation tool in this study. In brief, the hind paws of the animals were dipped in the ink and they were allowed to walk along the wooden walking alley (8.2 × 42 cm) with one closed end and the white paper was placed at the floor. From the footprints, the following parameters were obtained: print length (PL): distance from the heel to the third toe, toe spread (TS): distance from the first to the fifth toe, and intermediate toe spread (ITS): distance from the second to the fourth toe. The mean distances of all measurements mentioned earlier were used to calculate the following factors (dynamic and static):

(1) $$\begin{array}{c} \text{Toe}\;\text{spread}\;\text{factor}\;\left(\text{TSF}\right)=\frac{\left(\text{OTS}-\text{NTS}\right)}{\text{NTS}}\\ \text{Intermediate}\;\text{toe}\;\text{spread}\;\text{factor}\;\left(\text{ITSF}\right)=\frac{\left(\text{OITS}-\text{NITS}\right)}{\text{NITS}}\\ \text{Print}\;\text{length}\;\text{factor}\;\left(\text{PLF}\right)=\frac{\left(\text{OPL}-\text{NPL}\right)}{\text{NPL}}. \end{array}$$

Then, these values were used to calculate the sciatic function index (SFI) by the following formula [16]:

(2) SFI=(−38.3×PLF)+(109.5×TSF)+(13.3I TSF)−8.8.

According to this calculation, the normal values of SFI were in the range between +11 and −11 and a value of −100 indicated the total impairment [17].

### 2.5. Determination of Sensory Function by Using von Frey Filament Test

A series of 10 von Frey filaments (0.1 Gr, 0.2 Gr, 0.4 Gr, 0.8 Gr, 1.0 Gr, 1.2 Gr, 1.5 Gr, 2.5 Gr, 3.6 Gr, and 4.0 Gr) were used to evoke paw withdrawal response. Testing starts with the lowest filament of the series and each hind paw was stimulated 5 times. The intensity of withdrawal responses of each filament in a 3-second stimulation duration was recorded [18].

### 2.6. Determination of Sensory Function by Using Hot Plate Test

The rats were gently dropped into a plastic box with a metal floor that was preheated to a certain temperature (hot plate) [19]. The time duration between the initiation of the hot plate exposure and the time which the paw was raised from the floor was recorded and considered as paw withdrawal latency. Each animal was measured foot withdrawal reflex 5 times per session, with time interval of 5 min between tests. The average paw withdrawal latency was calculated and used as index. Minimal and maximal cut-offs were assigned at 0.5 to 20 seconds, respectively.

### 2.7. Determination of Sciatic Nerve Conduction by Nerve Conduction Velocity (NCV)

In this experiment, the sciatic nerve conduction velocity was performed by modifying the measurement of conduction velocity in human. In brief, the sciatic nerve of the animal was stimulated and the response of gastrocnemius which was supplied by this nerve was recorded as nerve activity [20]. In brief, the rat was anesthetized with thiopental sodium via intraperitoneal injection. The right sciatic nerve of each rat was exposed and gently dissected away from the surrounding muscle tissue and the electrodes were placed at two different points. The stimulating electrode was placed at proximal site of the lesion whereas the recording electrode was place at distal site of the lesion. The sciatic nerve was stimulated using electrical stimuli at an interval of 0.5 ms and amplitudes between 50 and 2000 mV. The response latency was recorded. Then the location of recoding electrode was moved from the original site. The change of muscle response latency was then recorded. The nerve conduction velocity was calculated by dividing the distance obtained from the displacement of recording electrode with the change of muscle response latency which occurred as the result of the displacement of recording electrode.

### 2.8. Fasting Blood Glucose Level

Blood samples were taken from rat tails after overnight fasting. The fasting blood glucose level was monitored at the end of experiment by using ACCU-CHEK active.

### 2.9. Homogenate Preparation

At the end of experiment, homogenate of right sciatic nerve was prepared in 1 mL of 0.1 M phosphate buffer, pH 7.4. The obtained nerve homogenate was adjusted to 10% w/v and centrifuged with microcentrifuge (SIG 1-15PK) at 10,000 g, 4°C for 1 hour. The supernatant was harvested and processed for the estimation of biochemical parameters.

### 2.10. Biochemical Parameters Assessments

#### 2.10.1. Determination of Malondialdehyde (MDA) Level

Level of malondialdehye (MDA), a lipid peroxidation marker, was monitored by using thiobarbituric acid reacting substances (TBARS) assay. In brief, 100 *μ*L of sample was mixed with the solution containing 100 *μ*L of 8.1% (w/v) sodium dodecyl sulphate** (**Sigma-Aldrich), 750 *μ*L of 20% (v/v) acetic acid (Sigma-Aldrich) (pH 3.5), and 750 *μ*L of 0.8% thiobarbituric acid (TBA) (Sigma-Aldrich). The solution was heated in a water bath at 95°C for one hour and cooled immediately under running tap water. Then, 500 *μ*L of chilled water and 2500 *μ*L of butanol and pyridine (Sigma-Aldrich) [15 : 1 v/v] were added into each tube and mixed thoroughly with vortex (Vortex-Genie 2). Then, the solution was centrifuged at 800 ×g for 20 minutes. The upper layer was separated and absorbance was measured at 532 nm with spectrophotometer (GENESYS 20). 1,3,3-tetraethoxypropane (TEP) (Sigma-Aldrich) was used as in [21]. The level of MDA was expressed as U/mg·protein.

#### 2.10.2. Determination of Superoxide Dismutase (SOD) Activity

The determination of SOD activity was carried out via nitroblue tetrazolium (NBT) reduction assay. In this assay, the xanthine-xanthine oxidase system was used as a superoxide generator. In brief, the reaction mixture contained 20 *μ*L of sample and 200 *μ*L of reaction mixture consisting of 57 mM phosphate buffer solution (KH_2_PO_4_) (Sigma-Aldrich), 0.1 mM EDTA (Sigma-Aldrich), 10 mM cytochrome C solution (Sigma-Aldrich), and 50 *μ*M of xanthine solution (Sigma-Aldrich) and 20 *μ*L of xanthine oxidase solution (Sigma-Aldrich) (0.90 mU/mL) was prepared at 25°C. The optical density was determined at 415 nm with a UV-spectrophotometer (Pharmacia LKB-Biochrom4060). A system devoid of enzyme was served as the control and three parallel experiments were conducted [22]. SOD activity was expressed as U/mg·protein.

#### 2.10.3. Determination of Catalase (CAT) Activity

Nerve catalase activity was assessed based on the ability of the enzyme to break down H_2_O_2_. In brief, 10 *μ*L of sample was mixed with the reaction mixture which contained 50 *μ*L of 30 mM hydrogen peroxide (Sigma-Aldrich) (in 50 mM phosphate buffer, pH 7.0), 25 *μ*L of H_2_SO_4_ (Sigma-Aldrich), and 150 *μ*L of KMnO_4_ (Sigma-Aldrich). After mixing thoroughly, the optical density was measured at 490 nm using GENESYS 20 spectrophotometer. A system devoid of the substrate (hydrogen peroxide) was served as the control. The difference in absorbance per unit time was expressed as the activity. An amount of enzyme required to decompose 1.0 M of hydrogen peroxide per minute at pH 7.0 and 25° is regarded as one unit [23]. The value of CAT activity was expressed as U/mg·protein.

#### 2.10.4. Determination of Glutathione Peroxidase (GPx) Activity

This assay was performed based on the glutathione recycling method by using 5,5′-dithiobis (2-nitrobenzoic acid) (DTNB) (Sigma-Aldrich) and glutathione reductase (Sigma-Aldrich). According to this method, the reaction between DTNB and GSH gave rise to the generation of 2-nitro-5-thiobenzoic acid and GSSG. Since 2-nitro-5-thiobenzoic acid was a yellow colored product, GSH concentration could be determined by measuring absorbance at 412 nm. In brief, a mixture containing 20 *μ*L of sample and the reaction mixture consisting of 10 *μ*L of dithiothreitol (DTT) (Sigma-Aldrich) in 6.67 mM potassium phosphate buffer (pH 7), 100 *μ*L of sodium azide (Sigma-Aldrich) in 6.67 mM potassium phosphate buffer (pH 7), 10 *μ*L of glutathione solution, and 100 *μ*L of hydrogen peroxide was mixed thoroughly and incubated at room temperature for 5–10 minutes. Then, 10 *μ*L of DTNB (5,5-dithiobis-2-nitrobenzoic acid) was added and the optical density at 412 nm was recorded at 25°C over a period of 5 min by using UV-spectrophotometer (Pharmacia LKB-Biochrom4060). Activities were expressed as nmoles/min/mg lens protein [24]. GPx activity was expressed as U/mg·protein.

#### 2.10.5. Determination of Aldose Reductase (AR) Activity

Aldose reductase activity was evaluated using colorimetric method. An assay mixture containing 0.7 mL of phosphate buffer (0.067 mol), 0.1 mL of NADPH (Sigma-Aldrich) (25 × 10^−5^ mol), 0.1 mL of DL-glyceraldehyde (Sigma-Aldrich) (substrate, 5 × 10^−4^ mol), and 0.1 mL of nerve supernatant was prepared. Absorbance was recorded against a reference cuvette containing all other components except the substrate, DL-glyceraldehyde. The final pH of the reaction mixture was adjusted to pH 6.2. The determination was performed after adding the substrate or DL-glyceraldehyde by measuring the decrease in NADPH absorbance at 390 nm over a 4-minute period via a UV-spectrophotometer (Pharmacia LKB-Biochrom4060) [25]. The enzyme activity was expressed as nmol/min/mg.

### 2.11. Histopathology Study

After anesthesia with sodium pentobarbital (60 mg/kg BW), the animals were perfused with 0.9% normal saline. Sciatic nerve was collected and immersed sequentially for 24 h in 10% formalin. The frozen sample was immersed in a stainless steel container filled with optimal cutting temperature (OCT) compound; the container was selected to fit the size of the sample. The specimens were frozen rapidly and 5 *μ*m thick sections were made using cryostat.

### 2.12. Determination of Axonal Density

The nerve sample was stained with toluidine blue and the axon density in the lesion sciatic nerve was determined. In brief, the sections were stained with 0.1% toluidine blue for 10–30 s, rinsed with running water for about 5 min, and mounted with glycerin under a cover glass. Histological analysis was performed by light microscope and the density of axons was accessed by using Image Pro-plus 5.1 program.

### 2.13. Statistical Analysis

All parameters were compared using one-way analysis of variance (ANOVA). The post hoc test was used to identify specific mean differences. They were represented as mean ± standard error mean (mean ± SEM). Statistical analysis was carried out using SPSS version 15. Differences were considered significant at *P* value < .05.

## 3. Results

### 3.1. Effect of PWCG on Fasting Blood Glucose Concentration

**Figure 2:**
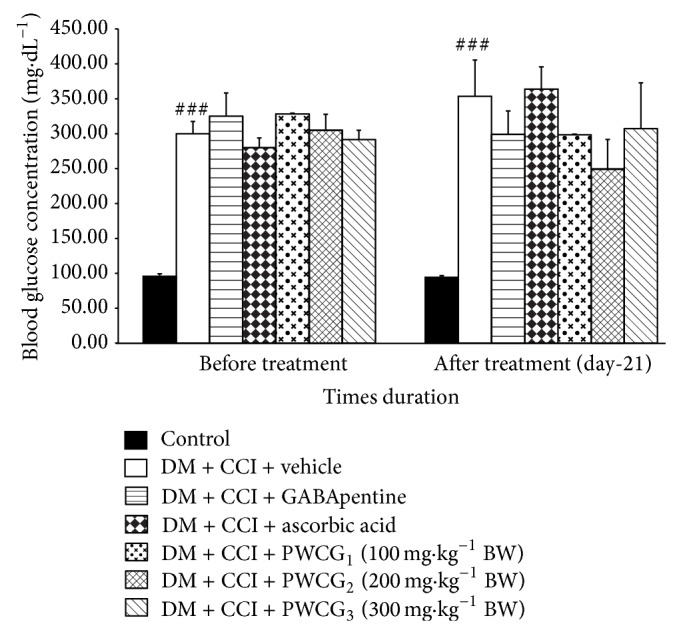
The effect of PWCG on fasting blood glucose concentration (*n* = 8/group). ^###^ *P* value < .001; compared to control rats.

The average fasting blood glucose levels of all the experimental groups were shown in Figure 2. It was found that the blood sugar levels of all diabetic rats were more than 250 mg·dL^−1^ throughout the study period. PWCG failed to produce a significant reduction of the fasting blood glucose level in all treated groups.

### 3.2. Effect of PWCG on Function Recovery in Peripheral Neuropathy in Diabetic Rats

Table 1 showed that, before CCI (baseline data), no significant changes among groups were observed. In addition, all groups still showed normal SFI. Diabetic rats plus CCI which received vehicle showed the significant reduction of SFI (*P* value < .001 all, compared to control group) and showed the impaired SFI throughout the study period. Diabetic rats which are subjected to CCI and received GABApentin failed to produce the significant change of SFI throughout the study period. Ascorbic acid significantly improved SFI of diabetic rats which are subjected to CCI at 9 and 12 days of treatment (*P* value < .05 and .01, resp.; compared with diabetic rats plus CCI which received vehicle). PWCG at dose of 100 and 200 mg·kg^−1^ BW significantly improved SFI of diabetic rats which are subjected to CCI at 6 (*P* value < .01 all, compared with diabetic rats plus CCI which received vehicle), 9 (*P* value < .05 and .01, resp., compared with diabetic rats plus CCI which received vehicle), and 12 days of treatment (*P* value < .01 and .05, resp., compared with diabetic rats plus CCI which received vehicle). It was found that diabetic rats plus CCI which were orally given PWCG at dose of 300 mg·kg^−1^ BW produced significant improvement in SFI since day 3 to day 12 of treatment (*P* value < .05, .05, .001, and .05, resp., compared with diabetic rats plus CCI which received vehicle). No significant improvement of SFI was observed in any treatment groups at the other treatment duration.

The effect of PWCG on sensory function of sciatic nerve was also evaluated by both von Frey filament test and hot plate test and data were shown in Tables 2 and 3. In Table 2, it was found that no significant differences among groups were observed at baseline data. Diabetic rats which received CCI and were orally given vehicle showed the significant reduction in paw withdrawal threshold which was evaluated via von Frey filament test every 3 days throughout a 21-day experimental period (*P* value < .01, .05, .01, .01, .05, .05, and .05, resp., compared to control group). GABApentin significantly mitigated the decreased paw withdrawal threshold induced by CCI in diabetic rats since day 3 to day 15 of treatment (*P* value < .01, .05, .05, .01, and .05, resp., compared to diabetic rats plus CCI which received vehicle). Diabetic rats plus CCI which received ascorbic acid showed the significant increase in paw withdrawal threshold in response to mechanical stimuli in diabetic rats plus CCI only at 12 days of treatment (*P* value < .5, compared to diabetic rats plus CCI which received vehicle). Interestingly, PWCG at dose of 100 mg·kg^−1^ BW significantly mitigated the decrease of paw withdrawal threshold in response to mechanical stimuli induced by CCI in diabetic rats at 9 and 12 days of treatment (*P* value < .05 all, compared to diabetic rats plus CCI which received vehicle) whereas PWCG at doses of 200 and 300 mg·kg^−1^ BW produced the significant mitigation effect on the reduction of paw withdrawal threshold in response to mechanical stimuli induced by CCI in diabetic rats between day 9 and day 15 of treatment (*P* value < .05 all, compared to diabetic rats plus CCI which received vehicle).

Table 3 showed that the diabetic rats which are subjected to CCI and received vehicle also decreased paw withdrawal latency in response to temperature stimuli evaluated by hot plate test (*P* value < .001 all, compared to control group). Both diabetic rats plus CCI which received GABApentin and diabetic rats plus CCI which received ascorbic acid significantly mitigated the decreased paw withdrawal latency in response to temperature stimuli since day 3 to day 15 of treatments (*P* value < .01 and .05; .001 and .01; .01 all; .001 and .01; .05 all, resp., compared to diabetic rats plus CCI which received vehicle). Low dose of PWCG failed to modulate paw withdrawal latency throughout the study period. However, diabetic rats plus CCI which received medium dose of PWCG significantly mitigated the increased paw withdrawal latency from day 6 to day 12 of treatment (*P* value < .05 all, compared to diabetic rats plus CCI which received vehicle) while those which received high dose of PWCG showed the significant improvement of paw withdrawal latency between day 3 and day 12 of treatment (*P* value < .05 all, compared to diabetic rats plus CCI which received vehicle).

### 3.3. Effect of PWCG on Nerve Conduction Velocity

**Figure 3:**
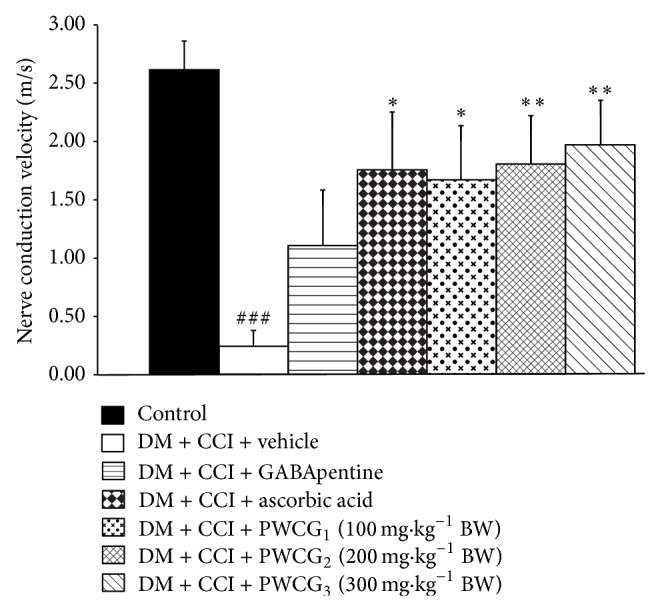
Effect of PWCG on sciatic nerve conduction velocity (*n* = 8/group). ^###^ *P* value < .001, compared to control group. ^∗, ∗∗^ *P* value < .05 and .01, respectively, compared to diabetic rats which were subjected to CCI and received vehicle.

The effect of PWCG on nerve conduction velocity was also evaluated. It was found that diabetic rats which are subjected to CCI significantly decreased sciatic nerve conduction velocity as shown in Figure 3. Diabetic rats which are exposed to CCI and received GABApentin failed to show the significant increase in NCV of sciatic nerve. Interestingly, diabetic rats plus CCI which received either ascorbic acid treatment or PWCG at the range used in this study significantly improved sciatic nerve conduction velocity (*P* value < .05, .05, .01, and .01, resp., compared with diabetic rats plus CCI which received vehicle).

### 3.4. Effect of PWCG on Oxidative Stress Markers and Aldose Reductase Activity

**Figure 4:**
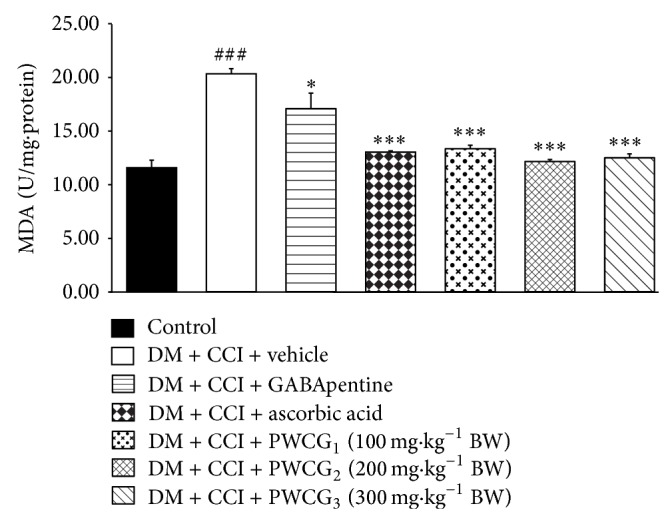
Effect of PWCG on malondialdehyde (MDA) level in the lesion sciatic nerve (*n* = 8/group). ^###^ *P* value < .001, compared to control group. ^∗, ∗∗∗^ *P* value < .05 and .001, respectively, compared to diabetic rats which were subjected to CCI and received vehicle.

**Figure 5 fig5:**
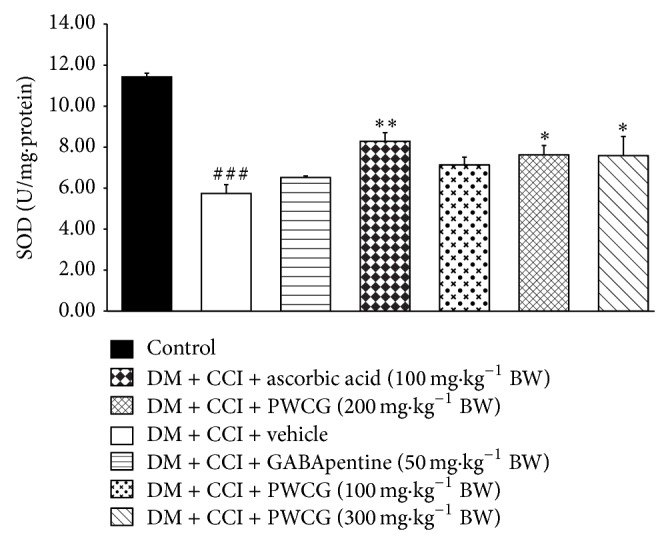
Effect of PWCG on superoxide dismutase (SOD) activity in the lesion sciatic nerve (*n* = 8/group). ^###^ *P* value < .001, compared to control group. ^∗, ∗∗^ *P* value < .05 and .01, respectively, compared to diabetic rats which were subjected to CCI and received vehicle.

**Figure 6 fig6:**
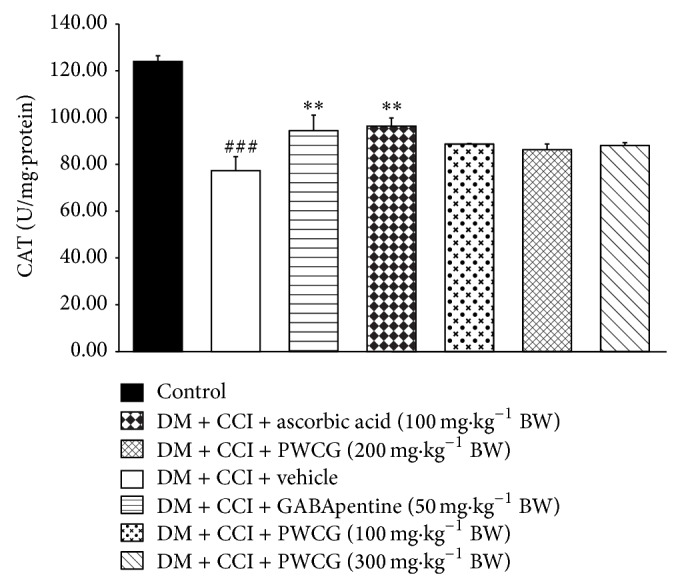
Effect of PWCG on catalase (CAT) activity in the lesion sciatic nerve (*n* = 8/group). ^###^ *P* value < .001, compared to control group. ^∗∗^ *P* value < .01, compared to diabetic rats which were subjected to CCI and received vehicle.

**Figure 7:**
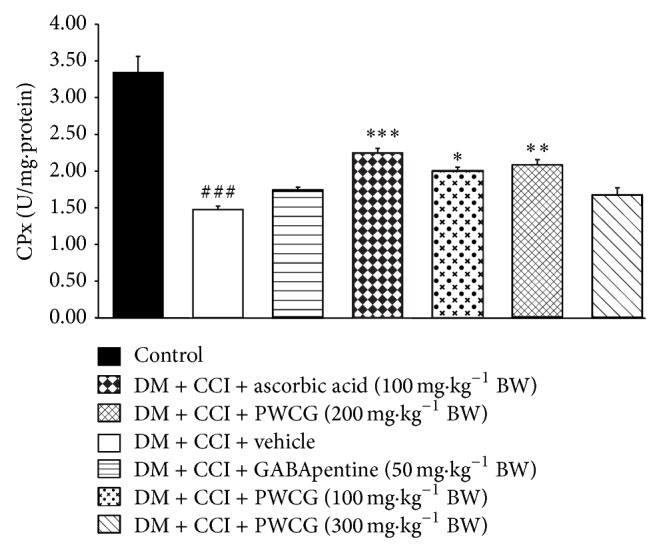
Effect of PWCG on glutathione peroxidase (GPx) activity in the lesion sciatic nerve (*n* = 8/group). ^###^ *P* value < .001, compared to control group. ^∗, ∗∗, ∗∗∗^ *P* value < .05, .01, and .001, respectively, compared to diabetic rats which were subjected to CCI and received vehicle.

Based on the previous findings that oxidative stress plays a crucial role in diabetic neuropathy, we also investigated the effect of PWCG on oxidative stress markers including the level of MDA and the activities of SOD, CAT, and GPx in the lesion nerve and results were shown in Figures 4 [Fig fig5] [Fig fig6]–7. It was found that diabetic rats plus CCI which received vehicle produced the significant reduction of MDA level in the lesion sciatic nerve (*P* value < .001, compared to control group). However, the activities of SOD, CAT, and GPx in diabetic rats plus CCI which received vehicle were markedly reduced (*P* value < .001 all, compared to control group). Diabetic rats plus CCI which received GABApentin significantly decreased MDA level but increased CAT activity in the lesion sciatic nerve (*P* value < .05 and .01, resp., compared to diabetic rats plus CCI which received vehicle). Ascorbic acid treatment could decrease MDA level but decreased SOD, CAT, and GPx activities in the lesion sciatic nerve of diabetic rats plus CCI (*P* value < .001, .01, .01, and .001, resp., compared to diabetic rats plus CCI which received vehicle). PWCG treatment at dose of 100 mg·kg^−1^ BW significantly decreased MDA level but increased GPx activity in the lesion sciatic nerve. Diabetic rats plus CCI which received PWCG at doses of 200 and 300 mg·kg^−1^ BW produced significant changes of the reduction of MDA level and the increase of SOD activity in the lesion sciatic nerve (*P* value < .001 all and .05 all, resp., compared to diabetic rats plus CCI which received vehicle). In addition, diabetic rats plus CCI which received the medium dose of PWCG also decreased GPx activity in the lesion sciatic nerve (*P* value < .01, compared to diabetic rats plus CCI which received vehicle).

**Figure 8:**
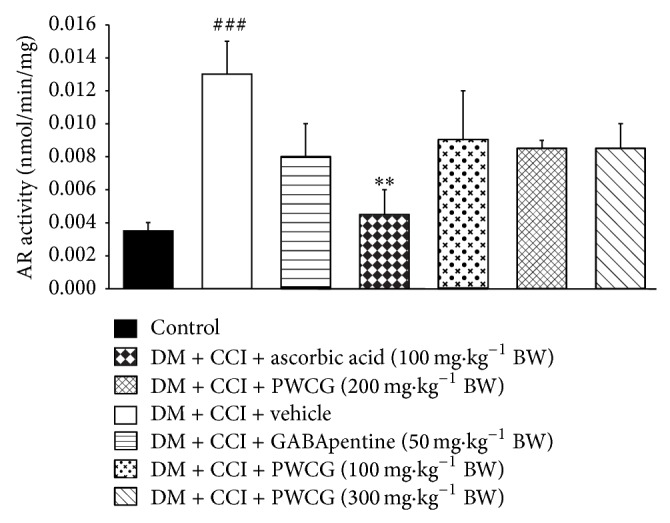
Effect of PWCG on aldose reductase (AR) activity in the lesion sciatic nerve. (*n* = 8/group) ^###^ *P* value < .001, compared to control group. ^∗∗^ *P* value < .01, compared to diabetic rats which were subjected to CCI and received vehicle.

The effect of PWCG on aldose reductase, a rate limiting enzyme in polyol pathway, was also investigated and data were shown in Figure 8. Diabetic rats plus CCI which received vehicle markedly increased aldose reductase activity in the lesion sciatic nerve (*P* value < .001, compared to control group). The only significant reduction of aldose reductase activity in the lesion sciatic nerve was observed only in ascorbic acid treatment group (*P* value < .01, compared to diabetic rats plus CCI which received vehicle). No other groups showed the significant change of this parameter.

### 3.5. Effect of PWCG on Density of Axon

**Figure 9:**
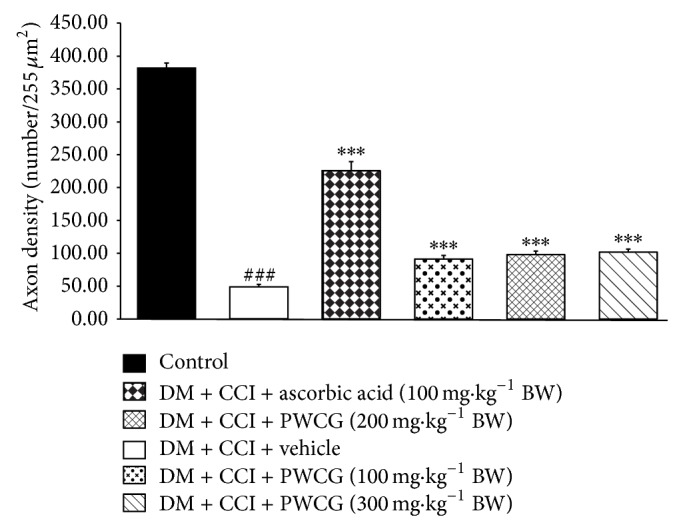
Effect of PWCG on the density of myelinated fibers evaluated by using toluidine blue stain (*n* = 8/group). ^###^ *P* value < .001, compared to control group. ^∗∗∗^ *P* value < .001, compared to diabetic rats which were subjected to CCI and received vehicle.

The effect of PWCG on density of myelinated axon in sciatic nerve evaluated by toluidine blue stain was investigated and results were shown in Figure 9. Diabetic rats plus CCI which were treated with vehicle significantly decreased density of myelinated axon (*P* value < .0001, compared to control group). Interestingly, ascorbic acid and all doses of PWCG used in this study mitigated the reduction of density of myelinated axon (*P* value < .001 all, compared to diabetic rats plus CCI which received vehicle).

## 4. Discussion

The current data has demonstrated that PWCG successfully improves the myelinated nerve fiber loss and nerve dysfunction in neuropathy with neuropathic pain in diabetic condition. PWCG also improves oxidative stress status in the lesion nerve while no suppression of aldose reductase activity is observed. In addition, no change of blood sugar was observed in PWCG treated rats.

Streptozotocin (STZ) can induce pancreatic beta cell toxicity and is often used to induce diabetes in experimental animals [26, 27]. STZ, a nitrosourea analogue, enters the pancreatic beta cell by using glucose transporter type 2 (GLUT 2) and produces the toxicity mainly via alkylation method. STZ alkylates DNA and protein in the cells leading to cell death. In addition to the alkylation, STZ can also produce oxidative stresses such as superoxide and hydroxyl radicals and nitric oxide (NO) which in turn induce beta cell destruction. Due to the decreased survival beta cell in pancreas, insulin secretion was also decreased leading to the decreased glucose uptake into peripheral tissues and finally resulting in hyperglycemia [28]. The results obtained from this study are also in agreement with the previous studies. Although the reduction of blood glucose can also improve neuropathy, no reduction of blood glucose was observed in PWCG treated groups. Therefore, the improved neuropathy in this study might not relate to this mechanism.

It has been reported that the pathophysiology of diabetic neuropathy involves the interplay of hyperglycemia, ischemia, and oxidative stress. The increased ischemic condition and oxidative stress status can exacerbate morphological changes of peripheral nerve in diabetic rat [29] and reduce the time duration to induce the damage of peripheral nerve and the clinical manifestation development. Therefore, the ischemic condition of nerve by using CCI in STZ-diabetic rats was performed [14]. Based on the previous finding, it has been reported that oxidative stress and the accumulation of sorbitol via the enhanced polyol pathway are responsible for the development of peripheral diabetic neuropathy [30, 31]. In this study, CCI can develop the impairment of motor function especially walking pattern together with the hyperalgesia conditions which are in agreement with the clinical manifestation observed in patients with diabetic neuropathy [32, 33].

Oxidative stress and the accumulation of sorbitol via the enhanced polyol pathway are reported to be responsible for the development of peripheral diabetic neuropathy [30, 31]. Both oxidative stress and sorbitol can damage the myelin sheath leading to the myelin degradation and the decreased NCV. In addition, both factors can decrease the myelinated nerve fiber density. It has been well known that myelinated fibers play the important role in the transmission of proprioception information which in turn plays the crucial role in postural stability. The abnormal gait which reflects the postural instability observed in this study may be due to the loss of myelinated fiber resulting in the decreased myelinated nerve density which in turn disturbs gait stability as shown by the impairment of SFI. Although hyperglycemia has been previously reported to be the important cause of nerve dysfunction, our data show that PWCG improve nerve dysfunction in diabetic condition without the change of hyperglycemic. Our finding is in agreement with previous study which showed that the nerve dysfunction is associated with the decreased myelinated nerve fiber density [31, 34].

Accumulative lines of evidence also demonstrate that oxidative stress plays an important role in neuropathic pain [35–39]. In addition, neuropathic pain can be attenuated by substances possessing antioxidant activity [34, 37]. Since the data obtained from our study showed that PWCG significantly increased SOD and GPx activities which in turn decreased the oxidative stress reflecting by the decreased MDA level, therefore we suggested that the antihyperalgesic effect of PWCG might occur partly via the decreased oxidative stress which in turn decreased the damage of peripheral nerve fiber resulting in the decreased pain fiber stimulation from potassium ion and prostaglandin which had been released from nerve injury. In addition to oxidative stress, neuropathic pain is also under the influence of many factors including polyol pathway [39]. The increased polyol pathway activity can induce nerve damage directly via the accumulation of sorbitol, a toxic substance, or indirectly via the increased oxidative stress. However, our data failed to show the suppression of aldose reductase in the lesion nerve. Therefore, the improved hyperalgesia in diabetic rats plus CCI which received PWCG might not be associated with the suppression of aldose reductase.

The inhibition of *γ*-aminobutyric acid or GABA pathway also plays a crucial role in modulating the response of peripheral nerve injury [40]. It has been reported that the activity of GABA pathway is decreased in neuropathic pain condition [41]. Therefore, the improved GABA activity might play a role in the improved neuropathic pain. However, it has been reported that quercetin, the main flavonoids in PWCG, was the GABA antagonist so it was less likely to produce antineuropathic pain via the increased GABA activity [42]. Unfortunately, the determination of GABA activity had not been performed in this study. Therefore, this was the limitation of our study and required further study to confirm the exclusion of the modulation of GABA pathway by PWCG.

In this study, no dose dependent manner of PWCG was observed. The possible explanation might be due to the masking effect of the other ingredients and the interaction effect among the ingredients on the effect of active ingredient (s). In addition, PWCG might not exert the effect directly on the observed parameters but it might exert the modulation effect via other mediators such as the signal molecules.

Taking all data together, PWCG might increase SOD and GPx activities which in turn decreased oxidative stress status in the lesion nerve giving rise to the improved nerve damage leading to the increased myelinated nerve fiber density and the improved SFI. In addition, the decreased nerve damage due to the decreased oxidative stress also decreased the stimulation of pain fiber leading to the improved paw withdrawal threshold intensity in response to mechanical stimuli and the improved paw withdrawal latency in response to thermal stimuli. However, another mechanism such as the involvement of GABA pathway may also play a role in the improvement of hyperalgesia and this requires further investigation. In addition, the improved oxidative stress might improve the damage of myelin sheath giving rise to the increased NCV of sciatic motor function. Although PWCG mitigated the neuropathy in diabetic condition, no reduction of blood glucose or the suppression of aldose reductase activity in the lesion nerve was observed. Therefore, both hypoglycemic effect and the suppression of polyol pathway may not be involved in antineuropathy effect of PWCG.

## 5. Conclusion

PWCG is the potential candidate to serve as functional food to improve peripheral neuropathy in diabetic patient. However, further researches about the pharmacokinetics and interactions with diabetic drugs are very much necessary before serving as the adjuvant therapy.

## Figures and Tables

**Table 1:** Effect of PWCG on the sciatic function index evaluated by walking track analysis (*n* = 8/group).

Time	Sciatic function index (SFI)						
	Control	DM + CCI + vehicle	DM + CCI + GABApentin (50 mg·kg^−1^ BW)	DM + CCI + ascorbic acid (100 mg·kg^−1^ BW)	DM + CCI + PWCG (100 mg·kg^−1^ BW)	DM + CCI + PWCG (200 mg·kg^−1^ BW)	DM + CCI + PWCG (300 mg·kg^−1^ BW)
Baseline	−9.29 ± 3.51	−10.49 ± 3.80	−7.40 ± 4.56	−8.25 ± 1.82	−8.65 ± 2.95	−10.56 ± 3.89	−7.59 ± 6.86
Day 1	−7.98 ± 1.35	−91.72 ± 1.58^###^	−84.36 ± 7.12	−87.93 ± 2.04	−85.03 ± 2.45	−90.76 ± 1.69	−88.86 ± 2.21
Day 3	−7.86 ± 2.00	−91.94 ± 1.36^###^	−90.59 ± 1.75	−86.28 ± 1.93	−89.02 ± 2.09	−90.45 ± 1.46	−80.81 ± 3.79^∗^
Day 6	−7.74 ± 1.73	−91.82 ± 1.48^###^	−89.86 ± 2.28	−86.28 ± 1.93	−83.69 ± 1.82^∗∗^	−83.56 ± 1.54^∗∗^	−84.93 ± 2.31^∗^
Day 9	−9.98 ± 1.89	−91.53 ± 1.77^###^	−91.72 ± 1.58	−86.73 ± 1.79^∗^	−86.54 ± 2.22^∗^	−84.41 ± 0.62^∗∗^	−83.54 ± 1.71^∗∗∗^
Day 12	−9.78 ± 2.40	−92.19 ± 1.11^###^	−90.24 ± 2.05	−84.32 ± 1.79^∗∗^	−84.01 ± 2.17^∗∗^	−87.14 ± 1.50^∗^	−86.38 ± 1.94^∗^
Day 15	−6.75 ± 2.54	−91.53 ± 1.77^###^	−91.89 ± 1.41	−85.50 ± 1.57	−89.65 ± 5.21	−90.56 ± 4.40	−85.52 ± 1.76
Day 18	−7.19 ± 1.37	−91.69 ± 1.61^###^	−90.88 ± 2.42	−82.42 ± 9.04	−93.91 ± 5.52	−84.20 ± 1.52	−88.24 ± 2.00
Day 21	−7.48 ± 1.75	−91.53 ± 1.77^###^	−89.15 ± 2.74	−81.45 ± 10.32	−89.85 ± 3.63	−83.03 ± 2.37	−87.57 ± 1.88

^###^ *P*-value <.001; compared to control rats.

^∗,∗∗,∗∗∗^ *P*-value <.05, .01 and .001 respectively; compared to DM rats with CCI which received vehicle.

**Table 2:** Effect of PWCG on paw foot withdrawal threshold intensity evaluated by von Frey filament test. (*N* = 8/group) ^#,##^ *P*-value <.05 and .01 respectively; compared to control rats. ^∗,∗∗^ *P*-value <.05 and .01 respectively; compared to DM rats with CCI which received vehicle.

Time	Paw withdrawal threshold intensity (g)						
	Control	DM + CCI + vehicle	DM + CCI + GABApentin (50 mg·kg^−1^ BW)	DM + CCI + ascorbic acid (100 mg·kg^−1^ BW)	DM + CCI + PWCG (100 mg·kg^−1^ BW)	DM + CCI + PWCG (200 mg·kg^−1^ BW)	DM + CCI + PWCG (300 mg·kg^−1^ BW)
Baseline	3.92 ± 0.08	3.92 ± 0.08	3.92 ± 0.08	3.92 ± 0.08	3.95 ± 0.05	3.95 ± 0.05	3.90 ± 0.02
Day 3	3.92 ± 0.08	3.02 ± 0.32^##^	3.84 ± 0.10^∗∗^	3.70 ± 0.30	3.61 ± 0.17	3.58 ± 0.24	3.66 ± 0.08^∗^
Day 6	3.84 ± 0.10	2.70 ± 0.56^#^	3.84 ± 0.10^∗^	3.70 ± 0.30	3.54 ± 0.30	3.31 ± 0.3	3.40 ± 0.13
Day 9	4.00 ± 0.00	2.80 ± 0.56^##^	3.84 ± 0.10^∗^	3.50 ± 0.50	3.66 ± 0.18^∗^	3.71 ± 0.18^∗^	3.71 ± 0.11^∗^
Day 12	3.92 ± 0.08	2.90 ± 0.48^##^	3.84 ± 0.10^∗∗^	3.70 ± 0.30^∗^	3.61 ± 0.17^∗^	3.71 ± 0.18^∗^	3.71 ± 0.09^∗^
Day 15	3.92 ± 0.08	3.10 ± 0.37^#^	3.92 ± 0.08^∗^	3.70 ± 0.30	3.66 ± 0.22	3.76 ± 0.23^∗^	3.71 ± 0.10^∗^
Day 18	4.00 ± 0.00	3.12 ± 0.49^#^	3.92 ± 0.08	3.70 ± 0.30	3.48 ± 0.22	3.53 ± 0.23	3.53 ± 0.10
Day 21	4.00 ± 0.00	3.24 ± 0.31^#^	3.84 ± 0.10	3.70 ± 0.30	3.54 ± 0.30	3.61 ± 0.17	3.71 ± 0.18

**Table 3:** Effect of PWCG on paw foot withdrawal threshold intensity evaluated by hot plate test test. (*N* = 8/group). ^###^ *P*-value< .001; compared to control rats. ^∗,∗∗,∗∗∗^ *P*-value <.05, .01 and .001 respectively; compared to DM rats with CCI which received vehicle.

Time	Paw withdrawal latency (sec)						
	Control	DM + CCI + vehicle	DM + CCI + GABApentin (50 mg·kg^−1^ BW)	DM + CCI + ascorbic acid (100 mg·kg^−1^ BW)	DM + CCI + PWCG (100 mg·kg^−1^ BW)	DM + CCI + PWCG (200 mg·kg^−1^ BW)	DM + CCI + PWCG (300 mg·kg^−1^ BW)
Baseline	4.47 ± 0.33	4.53 ± 0.53	4.60 ± 0.38	4.40 ± 0.32	4.54 ± 0.41	4.25 ± 0.26	4.21 ± 0.36
Day 3	4.20 ± 0.25	1.40 ± 0.16^###^	2.87 ± 0.17^∗∗^	2.40 ± 0.07^∗^	2.00 ± 0.15	2.17 ± 0.14	2.50 ± 0.51^∗^
Day 6	4.86 ± 0.29	1.60 ± 0.13^###^	2.70 ± 0.32^∗∗∗^	2.47 ± 0.23^∗∗^	1.87 ± 0.18	2.21 ± 0.14^∗^	2.17 ± 0.09^∗^
Day 9	4.20 ± 0.23	1.70 ± 0.13^###^	2.93 ± 0.07^∗∗^	2.73 ± 0.30^∗∗^	2.33 ± 0.34	2.42 ± 0.14^∗^	2.46 ± 0.20^∗^
Day 12	4.07 ± 0.40	1.33 ± 0.18^###^	2.66 ± 0.21^∗∗∗^	2.40 ± 0.29^∗∗^	1.90 ± 0.16	2.00 ± 0.22^∗^	2.04 ± 0.10^∗^
Day 15	3.53 ± 0.45	1.53 ± 0.20^###^	2.33 ± 0.15^∗^	2.27 ± 0.13^∗^	1.46 ± 0.13	1.67 ± 0.14	1.92 ± 0.10
Day 18	4.33 ± 0.40	1.40 ± 0.13^###^	1.93 ± 0.12	1.90 ± 0.14	1.33 ± 0.13	1.58 ± 0.12	1.67 ± 0.13
Day 21	4.40 ± 0.45	1.60 ± 0.13^###^	2.27 ± 0.19	2.09 ± 0.21	1.38 ± 0.17	1.46 ± 0.15	1.50 ± 0.15

## Abbreviations

DN: Diabetic neuropathy
PWCG: The combination extract of purple waxy corn and ginger
*Z. officinale*: * Zingiber officinale*
*Z. mays*: * Zea mays*
STZ: Streptozotocin
CCI: Chronic constriction injury
SFI: Sciatic function index
PWTI: Paw withdrawal threshold intensity
PWL: Paw withdrawal latency
NCV: Nerve conduction velocity
AR: Aldose reductase
mg: Milligram
kg: Kilogram
BW: Body weight
PL: Print length
TS: Toe spread
ITS: Intermediate toe spread
TSF: Toe spread factor
mV: Millivolt
MDA: Malondialdehyde
SOD: Superoxide dismutase
CAT: Catalase
GPx: Glutathione peroxidase
QE: Quercetin equivalent
TBARS: Thiobarbituric acid reacting substances
NBT: Nitroblue tetrazolium
U: Unit
mL: Milliliter
mM: Millimole
dL: Deciliter
GABA: *γ*-Aminobutyric acid.
